# Mobile Usage Duration and Usability of Mobile Health Applications Among Older Adults in Saudi Arabia: A Usability-Centered Model Informed by Technology Acceptance Theory

**DOI:** 10.3390/healthcare14131957

**Published:** 2026-07-02

**Authors:** Tarfah Aldabban, Manjur Kolhar, Fajr Alabdullah, Safa Abbas Alhaddad, Shahad Alharbi

**Affiliations:** Department of Health Information Management and Technology, College of Applied Medical Sciences, King Faisal University, Al-Ahsa 31982, Saudi Arabia; 222425999@student.kfu.edu.sa (T.A.); 222430492@student.kfu.edu.sa (F.A.); 222415273@student.kfu.edu.sa (S.A.A.);

**Keywords:** mobile health, mHealth, usability, older adults, technology acceptance model, user satisfaction, mobile usage duration, digital health adoption

## Abstract

**Background:** With the vast and fast-growing number of mHealth applications supporting health, disease management and self-care for older people, the usability of these applications has become a critical factor determining their acceptance and usage. In order to develop mHealth applications suitable for the aging population, it is important to investigate the relationship between older people’s experience with mobile technology in the past, their perception of the usability of mHealth applications and their subsequent use of these applications. **Objective:** This study investigated the impact of the length of mobile usage on the perceived mHealth application usability of older adults, and the impact of mHealth application usability on the mHealth application user satisfaction and frequency of use of older adults. **Methods:** This study is based on a cross-sectional survey among older individuals in Al-Ahsa, Saudi Arabia. The measurement model consisted of five distinct constructs with fifteen corresponding indicators including efficiency, learnability, memorability, error handling, and user satisfaction. In terms of analysis, this study included reliability and descriptive statistics as well as correlation and regression analysis, as well as simple and bootstrapped mediation analysis, and, finally, confirmatory factor analysis (CFA) and structural equation modeling (SEM). Based on discriminant validity, the findings suggest that four first-order dimensions, efficiency, learnability, memorability, and error handling, constitute second-order usability dimensions. **Results:** A total of 271 older adults were included in the final analysis. All constructs demonstrated satisfactory reliability and convergent validity, with Cronbach’s alpha values ranging from 0.797 to 0.862, Composite Reliability values ranging from 0.798 to 0.860, and Average Variance Extracted values ranging from 0.568 to 0.673. Structural equation modeling revealed that mobile usage duration significantly influenced usability (β = 0.616, *p* < 0.001), usability significantly influenced user satisfaction (β = 0.953, *p* < 0.001), and user satisfaction significantly influenced use frequency (β = 0.193, *p* = 0.002). The second-order structural model demonstrated excellent fit to the data (χ^2^/df = 1.824, CFI = 0.972, TLI = 0.966, GFI = 0.940, AGFI = 0.928, RMSEA = 0.055). **Conclusions:** Usability plays a central role in explaining the satisfaction of older people with mHealth services and their continuous use of applications. Older people’s experience with their smartphones is associated with their perceptions of the usability of mHealth applications. Higher perceived usability of mHealth applications is positively associated with greater user satisfaction and more frequent use of these applications among older adults. The findings are in line with a usability-centered technology acceptance model. Design of mHealth services should be based on user-centered design principles. In addition to other design principles, efficiency, learnability, memorability, error handling and other usability principles should be particularly addressed in order to increase acceptance of mHealth services by older people.

## 1. Introduction

With an accelerating global shift in the demographics of populations, older people are becoming an increasingly larger proportion of the global community [1,2,3,4,5]. According to available projections, by 2030, more than one in six of the world’s population is expected to be aged 60 years and older [6,7,8]. While this large elderly population is still far from Saudi Arabia, the size of the older population in the country is increasing steadily, representing about 6 per cent of the population in 2022 [9]. This aging population faces a high prevalence of chronic health conditions, and many individuals aged 65 years and older live with two or more health conditions [10]. Saudi Vision 2030 aims to raise the life expectancy to 80 years by 2030 and utilizing digital health technologies such as mobile health (mHealth) applications can play a pivotal role in connecting the older adult with the healthcare system and managing chronic diseases [11,12,13,14,15,16,17,18,19,20,21].

Although there are potential benefits and widespread availability of mHealth services, they have yet to be adopted by older adults [22,23,24,25,26]. Despite awareness of the availability and benefits of mHealth among older adults, their usage rates are very low [27]. Studies in Saudi Arabia have found that older adults perceive mHealth applications as being difficult to use and often lack digital literacy skills [27]. Furthermore, cognitive, vision, and hearing declines associated with aging can render many mHealth applications inaccessible [24,28]. Older adults also worry about making errors when using mobile technology [29,30]. Despite the support of family members and friends, most older adults report needing help to use mHealth applications, which calls for the development of more user-friendly applications designed specifically for older adults.

When exploring older adults’ acceptance of information technology, traditional frameworks, such as the technology acceptance model (TAM), might not be sufficient [31,32,33,34,35]. The TAM posits that users’ perceived usefulness and perceived ease of use determine their actual technology use. However, when addressing special user groups, as the older adults in this study are, a more comprehensive view on usability might also be necessary [31,32,33,34,35]. This paper therefore extends the application of the TAM with additional usability dimensions. For instance, the efficiency dimension of usability has been used as an indicator for perceived usefulness. Other dimensions like learnability, memorability, error handling and satisfaction complement perceived ease of use and measure users’ experience and confidence.

In addition to the self-efficacy concept, this study incorporates mobile usage duration as an external variable. Increased exposure to smartphone technology is expected to enhance users’ familiarity with mobile interfaces and improve their usability perceptions. However, increased exposure may not improve all usability dimensions equally, in particular, the error factor.

While there is an increasing body of research on mHealth adoption, there is little empirical research that specifically examines the relationship between older adults’ smartphone usage behavior and their perceptions of specific usability aspects, particularly within the Middle Eastern region. Moreover, although usability is seen as a critical determinant that explains how older adults experience mobile usage and, consequently, influence their satisfaction and continued use of the technology, it has received scant attention in prior research. This study investigates the relationship between the duration of mobile usage and the usability of mobile health applications by older adults. It further explores the relationship between application usability, user satisfaction, and ongoing use, within the context of older adults in Al-Ahsa, Saudi Arabia.

Despite the great potential of mHealth in supporting healthy aging and in chronic disease self-management, there is a dearth of studies that have explored mHealth adoption by older adults in Saudi Arabia. While a few studies have been conducted in Saudi Arabia to understand the perspectives of older adults and patients with chronic diseases towards digital health in general, to identify barriers to the use of mHealth services, and to investigate the intentions of healthy individuals and patients with chronic diseases to use mHealth services, the majority of the international studies that have explored mHealth adoption by older adults have used accepted technology acceptance models or have been qualitative studies that explored the barriers to the use of mHealth services by older adults. Most of these studies have also focused on only one dimension of usability. Most research on technology acceptance uses well-established constructs to explain users’ behavior such as perceived usefulness, perceived ease of use and users’ intention to use a system. However, in order to understand the usability of a system, it is more important to understand how users perceive the usability of a system on the basis of their experience with similar technology. In the context of this research, experience with smartphones, in particular, the duration of time that users use mobile phones, is important. While previous research has dealt with the experience of users with technology in general, there is a specific gap in the research when it comes to determining the mHealth usability of older adults.

There is a considerable gap in the research examining the relationship between smartphone experience and perceived usability of mHealth applications among older adults. Furthermore, limited research has investigated the associations between perceived usability, user satisfaction, and the frequency of mHealth application use. The current study is intended to address this gap. A novel usability-centered framework was developed and validated. The framework views usability as a second-order construct consisting of four first-order constructs of efficiency, learnability, memorability and error handling. Unlike prior studies of mHealth that focused on users’ beliefs about adopting using information technology or their intentions to use mHealth, the current study explored the relationships between the experience of using smartphones, overall usability of mHealth, users’ level of satisfaction with mHealth and the frequency with which users use mHealth. This is the first study conducted in Saudi Arabia that investigated usability as a higher-order construct and explored the role this construct plays in users’ continued engagement with mHealth services.

## 2. Conceptual Framework and Hypothesis Elaboration

The proposed conceptual framework integrates usability theory with the findings from technology acceptance research. Within this framework, usability [36,37,38,39] is described as a higher-order construct that is measured by four first-order dimensions: efficiency, learnability, memorability, and error handling. Efficiency, learnability, memorability, and error handling are widely recognized dimensions of usability that influence users’ experiences and interactions with digital systems. This conceptualization is rooted in usability theory as well as the results of the measurement model, which demonstrated interdependencies between the four mentioned dimensions of usability. The framework proposes a positive relationship between prior smartphone experience and older adults’ perceptions of the usability of mHealth applications. In turn, perceived usability is positively associated with user satisfaction, which is positively associated with the continued use of mHealth applications.

Hypotheses:

**H1.**  *Mobile usage duration is positively associated with overall usability of mHealth applications.*

**H2.**  *Overall usability is positively associated with user satisfaction.*

**H3.**  *User satisfaction is positively associated with the frequency of mHealth application use.*

**Figure 1:**
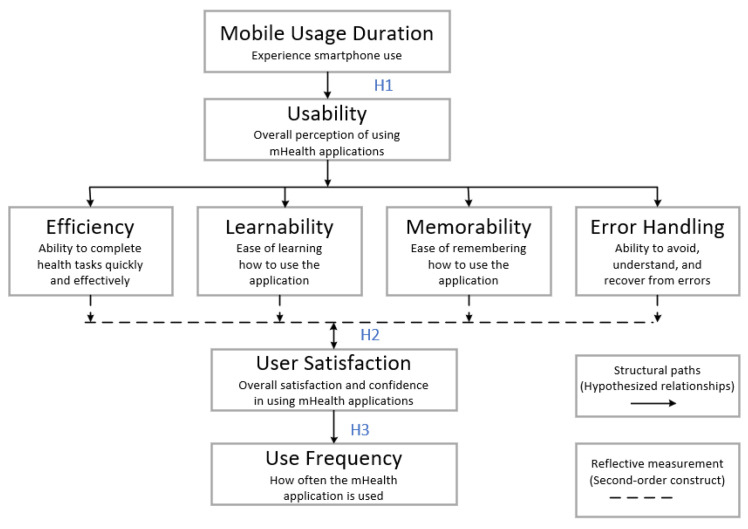
Conceptual framework.

The conceptual framework (see Figure 1) positions mobile usage duration as the key antecedent variable. Users with greater mobile usage duration have increasing experience of using mobile devices. We measured mobile usage duration by mobile usage experience rather than by mHealth application usage. Thus, an older adult with longer mobile usage duration (i.e., more smartphone experience) becomes more familiar with various mobile interface types, navigation, and functions to be used on a smartphone, as well as touch and other features that can be used on a smartphone. This experience can help to decrease technology-related barriers, and subsequently to increase the perceived usability value of mHealth applications. In particular, they become familiar with using mobile interfaces, navigating through them, and interacting with features. As a result, they would perceive mHealth applications as more usable.

The conceptual framework illustrating these relationships is presented in Figure 1.

The concept of usability in the context of this work model will be of a second-order latent construct, which is described by four dimensions of first-order latent constructs, i.e., efficiency, learnability, memorability and error handling. Efficiency describes the speed of a user’s completion of health-related tasks within the application in an accurate fashion. Learnability describes how easy it is for users to learn to use the application. Memorability assesses how easily users can remember how to use the application after an extended period of inactivity. Error handling describes how users can avoid errors, understand errors made and recover from errors in an operational context within the application. The framework builds on three hypotheses. Firstly, higher smartphone experience will translate into higher smartphone usability (H1). Higher usability in turn will increase user satisfaction (H2). User satisfaction assesses users’ overall experience when using mHealth applications. It can be described by terms such as comfort, trust, and overall satisfaction. Finally, higher user satisfaction is expected to increase use frequency (H3), i.e., the number of times a user uses an mHealth application. This framework outlines a sequence of positive relationships among the study constructs. Firstly, older adults’ smartphone experience is positively related to their perceptions of the usability of mHealth applications. Secondly, perceived usability of mHealth applications is positively related to user satisfaction. Thirdly, user satisfaction is positively related to the continued use of mHealth applications. The higher-order construct of usability provides a parsimonious and theoretically grounded framework for examining the relationships among smartphone experience, perceived usability, user satisfaction, and continued use of mHealth applications among older adults.

## 3. Methods

### 3.1. Study Design and Setting

The aim of this study is to investigate the relationship between the duration of mobile use and the usability of mobile health applications and their effects on older adults. A cross-sectional survey was conducted in Al-Ahsa, Saudi Arabia, a place where there is a rapid transformation in digital health with many mobile-based healthcare services having been introduced. The survey instrument, including all measurement items used for the usability constructs, is provided in Appendix A.

### 3.2. Participants and Sampling

The study aims to investigate the needs and experiences of community-dwelling older adults residing in the Al-Ahsa region of Saudi Arabia. Based on the healthcare framework for Saudi Arabia, participants included individuals of 60 years and above. Measures for mobile usage duration and use frequency were taken from the survey pertaining to users’ behavior regarding the use of smartphones and mHealth applications. Measures for usability and satisfaction were based on multi-item 7-point Likert scales. Participants were recruited by means of non-probability convenience sampling via social media and messaging apps. Participants were directed to an online survey via a link that was posted on these platforms. Participants were able to take part in the study as and when they chose to by completing the online survey. A total of 384 surveys were distributed to potential participants, 113 of which were aged 50–59 years and thus were screened out of the study in accordance with the study’s age eligibility criteria. Thus, the study included 271 older adults (all of whom were 60 years of age or older), who represented 70.6% of all study participants who returned surveys. There were 78 (28.8%) participants aged 60–69 years, 108 (39.9%) participants aged 70–79 years, and 85 (31.4%) participants 80 years and older. Females comprised 53.9% (n = 146) of the sample and males comprised 46.1% (n = 125) of the sample. In terms of educational attainment, 107 (39.5%) of the participants were illiterate, 96 (35.4%) completed school and 68 (25.1%) held a university degree or higher. Table 1 describes the process for selecting participants for the study and the demographic characteristics of the final study sample. Due to the non-probability convenience sampling strategy used in this study, the findings will not be generalizable to the broader population of older adults in Saudi Arabia (see Table 1).

### 3.3. Data Collection Procedure

Data were collected by means of a bilingual online survey (in Arabic and English). Participants completed the online questionnaire either independently or with the assistance of a researcher. Before participating, respondents were informed about the objective of the study. They were advised that participation in the study was voluntary and that all information collected would be treated in the strictest confidence. They were also asked to agree to participate and to indicate their consent to the study by clicking a mouse on an “agree” button to access the online questionnaire. The questionnaire was divided into two sections. The first section dealt with demographic information as well as the characteristics of participants’ usage of their smartphones. The second section included participants’ perceptions of the usability of mHealth applications by means of five dimensions (efficiency, learnability, memorability, error handling and user satisfaction). All data were recorded anonymously. No personally identifiable information was collected and all data and information from participants were used in accordance with the above-mentioned ethical principles for research in order to guarantee the protection of the privacy of the participants as well as confidentiality and voluntary participation.

### 3.4. Measures

#### 3.4.1. Mobile Usage Duration

These variables assess participants’ exposure to smartphones in terms of the number of hours they typically use their smartphone in a day. General familiarity with smartphones in terms of the amount of time spent using them predicts usability perceptions. Specifically, users who use their smartphones more are more likely to have the ability to use applications, recognize basic smartphone user interface components, and complete tasks on smartphones. In addition, for older adult users, usability of mHealth applications must also consider the technology with which they are currently familiar and actively use, and for whom assessment of mHealth usability must take into account their ability to use current mHealth applications.

#### 3.4.2. Usability Constructs

To measure usability in this study, the following five criteria were tested: efficiency, learnability, memorability, errors and user satisfaction. Three questions were posed for each of the five criteria. All questions were assessed on a 7-point Likert scale, where 1 = ‘strongly disagree’ and 7 ‘strongly agree’. To assess efficiency, items asked whether users could complete health-related tasks within the application quickly and efficiently, and whether they were able to accomplish health-related goals with minimal effort and in a logically organized sequence. Efficiency of use is a priority for older adults because it can reduce cognitive effort while maximizing performance.

Learnability addresses the question of how typical users might learn basic features of an application to meet their needs. Learnability addresses issues like instructions for new users, learning basic features, and independent use of an application. The goal of high learnability is to have new users learn the basic features of an application quickly enough so that they can start using the application and then continue to use it over time.

For older adults, who do not use applications on a regular basis, memorability is especially important. How easy is it for users to remember how to use an application after several months of no use? How much relearning occurs when an individual comes back to an application after months or even years of inactivity? Errors. How many errors do users make using a product? How well does a product support users who make errors? Are users able to correct their own mistakes quickly? User experience/satisfaction with the system. How comfortable and confident do users find the tool easy to use? How satisfied users are with the overall application. Usability contributes to system outcomes. Several of the outcomes are identified as key outcome variables.

#### 3.4.3. Use Frequency

Frequency of use was measured by assessing participants’ behavioral engagement with mHealth applications. Specifically, we asked participants how often (on average) they use mHealth applications in a typical day/week. This question aimed to measure continued usage of participating mHealth applications and assess the degree to which users naturally incorporate them into their behavior.

Usability research has repeatedly demonstrated that use frequency is a reasonable surrogate for behavioral intention and system use. In practical evaluations, conducted over extended periods of time, use frequency is often used as an indicator of users’ acceptance of and actual use of an application for health-related tasks for which it is intended, and in this work, use frequency is used as the final outcome variable to measure the effect of usability and user satisfaction. The full survey, with all items used to measure the studied variables, is illustrated in Appendix A (Table A1). In addition, Appendix B includes normality tests and results (Table A2), regression results and multicollinearity tests (Table A3).

## 4. Results

### 4.1. Reliability Analysis

Before testing the proposed conceptual framework, we tested the internal consistency reliability of all multi-item constructs by calculating their Cronbach’s alpha coefficients. We conducted the reliability analysis by using data from a sample of 271 older adults, who were 60 years and older, as shown in Table 2.

All constructs demonstrated satisfactory levels of internal consistency, with Cronbach’s alpha values ranging from 0.797 to 0.862. Learnability exhibited the highest reliability (α = 0.862), followed by memorability (α = 0.856), satisfaction (α = 0.835), efficiency (α = 0.834), and error handling (α = 0.797). According to commonly accepted guidelines, Cronbach’s alpha values above 0.70 indicate acceptable reliability, while values above 0.80 indicate good reliability. Therefore, all constructs exceeded the minimum recommended threshold and were retained for subsequent analyses.

Each construct was measured using three questionnaire items. Although three-item scales represent the minimum acceptable specification for latent construct measurement, the obtained reliability coefficients indicate adequate internal consistency among the items within each construct. These findings suggest that the measurement scales consistently captured the intended usability and satisfaction dimensions of mobile health application usage among older adults.

The satisfactory reliability results provided empirical support for proceeding with descriptive analysis, correlation analysis, and the subsequent confirmatory factor analysis (CFA) and structural equation modeling (SEM) procedures used to evaluate the proposed second-order usability framework.

### 4.2. Descriptive Statistics

Table 3 summarizes the descriptive statistics for the study variables, for the final sample of 271 older adults. The mean values of the study variables range from 3.608 to 3.857 on a seven-point Likert scale. In general, the values indicate that the older adults in the study have a moderate perception of the usability and of the degree to which they are satisfied with the mobile health application that they use. The highest mean value for the measured variables was recorded by the satisfaction variable (M = 3.857, SD = 1.875). This variable was followed by the errors variable (M = 3.755, SD = 1.956), the learnability variable (M = 3.720, SD = 2.039), the memorability variable (M = 3.684, SD = 2.072), and the efficiency variable (M = 3.608, SD = 1.949). The high standard deviations for all the variables indicate that there is moderate variability in the responses provided by the participants. The medians for the constructs’ middle measures ranged from 3.333 to 3.667. The interquartile ranges (25th% to 75th%) within the constructs appeared to be spread out sufficiently to capture the participants’ range of responses. The lowest to highest values across all of the measures spanned the full range of the 7-point Likert scale. In other words, all of the values on the scale were used. The skewness values for the study variables ranged from 0.084 to 0.219 and the kurtosis values ranged from −1.438 to −1.325. These statistics are all within typical boundaries for normality, and the study variables are essentially normal in form. The small positive values for skewness indicate that there is a slight bias towards higher categories on the measures. The study variables are also somewhat flat in comparison to a normal distribution, with the negative values for kurtosis indicating that they are more spread out in the outer regions than would be expected under normality. Additional statistical details, including normality assessment, extended correlation matrices, regression diagnostics, and mediation results, are provided in Appendix B. The detailed normality statistics (skewness and kurtosis) are presented in Appendix B.

The univariate normality of the study variables was assessed by means of the Shapiro–Wilk test as well as by the skewness and kurtosis statistics. The results of the test for normality are displayed in Table 4. The Shapiro–Wilk test for all variables was significant on the 0.001 level, thus indicating a deviation from normality. However, the Shapiro–Wilk test is a very sensitive test, especially in cases of medium to large sample sizes (e.g., N = 271 in the present study). Thus, even a rather marginal deviation from normality can be detected by the test on a statistically significant level. However, this deviation might not have any substantial impact on the results of multivariate analyses. Skewness and kurtosis values for the study variables were examined for the degree of non-normality. The study variables showed a slight positive skewness (0.084 to 0.219). The kurtosis values for the study variables ranged from −1.438 to −1.325. Thus, the study variables were moderately platykurtic and therefore flatter than the normal distribution. However, all the skewness and kurtosis values were within the commonly accepted limits of |skewness| < 2 and |kurtosis| < 7 for approximate normality. Thus, the study variables were determined to be suitable for parametric statistics. The efficiency construct exhibited the highest level of skewness at 0.219, while memorability showed the least amount of skewness at 0.084. Similarly, memorability showed the lowest kurtosis at −1.438, and errors exhibited the highest kurtosis at −1.325. However, the differences between the various constructs were minimal. Overall, the summary statistics and tests of normality for the study’s variables revealed little evidence of serious departures from normality. Thus, the data were deemed suitable for use in a variety of parametric analyses, including correlation, regression, and structural equation modeling. However, in order to enhance the robustness of the model’s parameter estimates as well as the associated tests of statistical significance, used in the mediation model and the structural model.

### 4.3. Correlation Analysis

Correlations were calculated to assess the relationships between mobile usage, the usability dimensions, overall satisfaction and use frequency. The results are summarized in Table 5. Correlations for mobile usage were positive for all measures. Moderate positive correlations were found between mobile usage and the measures of efficiency (r = 0.63, *p* < 0.001), learnability (r = 0.58, *p* < 0.001), memorability (r = 0.59, *p* < 0.001), error handling (r = 0.55, *p* < 0.001), satisfaction (r = 0.52, *p* < 0.001), and use frequency (r = 0.39, *p* < 0.001). These correlations indicate that older adults with more experience with smartphones perceive mHealth applications as more usable and report greater satisfaction with mHealth applications and more use of them.

Correlations between all usability dimensions were positive and high. Efficiency was highly correlated with learnability (r = 0.82, *p* < 0.001), memorability (r = 0.86, *p* < 0.001), and error handling (r = 0.81, *p* < 0.001). Learnability was highly correlated with memorability (r = 0.84, *p* < 0.001) and error handling (r = 0.83, *p* < 0.001). Memorability and error handling were highly correlated (r = 0.82, *p* < 0.001). The results showed that the dimensions of usability were all interrelated. A CFA and subsequent SEM were then used to test the appropriateness of modeling these dimensions as indicators of some higher-order usability construct. This factor consists of efficiency, learnability, memorability, and error handling.

Correlations were also found between all of the usability dimensions and user satisfaction. User satisfaction ratings were found to be strongly associated with efficiency (r = 0.80, *p* < 0.001), learnability (r = 0.79, *p* < 0.001), memorability (r = 0.79, *p* < 0.001), and error handling (r = 0.75, *p* < 0.001). Therefore, older adults’ perceptions of usability of mHealth applications are all very positively associated with their satisfaction with using these applications. In additional bivariate analyses, use frequency was found to be weakly but positively correlated with several other variables: mobile usage (r = 0.39, *p* < 0.001), efficiency (r = 0.27, *p* < 0.001), learnability (r = 0.24, *p* < 0.001), memorability (r = 0.20, *p* < 0.001), and error handling (r = 0.24, *p* < 0.001). On the other hand, the correlation between use frequency and satisfaction was very weak and not significant (r = 0.11, *p* > 0.05). Apparently, high satisfaction does not translate into higher use frequency of mHealth applications by older adults in the bivariate correlation framework. However, correlation analysis merely assesses the existence of the relationship between two variables, while the strength of their relationship is estimated. Therefore, the results of the correlation analysis between these two variables should be compared with the results of the multivariate analysis (presented later in this study) where the relationship between these two variables is assessed while all other variables in the study are controlled for.

### 4.4. Structural Equation Modeling

The SEM analysis was conducted by using the semopy package in Python 3.14. The proposed second-order factor model was estimated by using the Maximum Likelihood (ML) method. In the model, a second-order factor, usability, was represented by four first-order factors, efficiency, learnability, memorability, and error handling. The structural relationships between mobile usage duration and four factors of usability, user satisfaction and use frequency were examined by using the standardized path coefficients, the standard errors, z-values, and *p*-values. Before conducting the SEM analysis, the data were checked for missing values and other data problems. All cases with missing values in the SEM variables were deleted using listwise deletion (dropna()) for a total of 271 cases (60+ years) for the analysis. Mediation effects were calculated in a supplementary analysis for the mediated effects by conducting a regression-based bootstrapping analysis using Python to compute indirect effects. Indirect effects were calculated by means of 5000 bootstrap resamples with replacement from the data, fixing a seed of 42 to the random number generator. The 95% bootstrap confidence intervals for the indirect effects were computed. The mediation was counted as significant if the resulting confidence intervals did not contain zero. To assess the model fit, several indicators of good fit were checked. This included the chi-square value (χ^2^) as well as the ratio of chi-square to degrees of freedom (χ^2^/df). Furthermore, the Comparative Fit Index (CFI), the Tucker–Lewis Index (TLI), the Goodness-of-Fit-Index (GFI), the Adjusted Goodness-of-Fit-Index (AGFI), the Root Mean Square Error of Approximation (RMSEA), the Akaike Information Criterion (AIC) as well as the Bayesian Information Criterion (BIC) were calculated. Three structural relationships among mobile usage duration, usability, user satisfaction, and use frequency were investigated. The construct usability was modeled as a second-order factor that is reflected by four first-order factors: efficiency, learnability, memorability, and error handling.

The structural path coefficients are presented in Table 6. Our findings show that for older adults, mobile usage duration had a significant positive effect on usability (β = 0.616, SE = 0.087, z = 10.374, *p* < 0.001). Thus, H1 was supported. This finding indicates that the more an older adult uses his/her mobile, the more he/she believes that mHealth applications are usable.

Usability had a very strong positive effect on user satisfaction (β = 0.953, SE = 0.078, z = 14.585, *p* < 0.001). This finding supports hypothesis H2 and is the strongest relation found in the model. Usability clearly is the main factor for user satisfaction with mHealth applications by older adults. The results of the study revealed a significant positive correlation between user satisfaction and use frequency with a beta value of (β = 0.193, SE = 0.024, z = 3.086, *p* = 0.002) supporting H3 of the study. Thus, it can be stated that a user who is satisfied with an mHealth application will use it more frequently. The fit of the second-order structural model was assessed through a number of indices that provide an overall assessment of how well a proposed model fits a set of data. As shown in Table 7, the second-order structural model fitted the data extremely well with χ^2^/df equal to 1.824, CFI equal to 0.972, TLI equal to 0.966, GFI equal to 0.940, AGFI equal to 0.928 and RMSEA equal to 0.055. Clearly, all of the major fit indices indicated that the proposed framework was an appropriate representation of the relationships between mobile usage duration, usability, user satisfaction and use frequency. For the purpose of model parsimony, we refer to the Akaike Information Criterion (AIC = 74.452) and the Bayesian Information Criterion (BIC = 211.333) for the proposed second-order model. In our view, the second-order model achieves an adequate tradeoff between explanatory power and model complexity.

### 4.5. Evaluation of the Measurement Model

Before we test the structural relationships within our data, we have to test the measurement model. The results of the confirmatory factor analysis (CFA) are displayed in the following four tables, where we checked for indicator reliability, internal consistency reliability, convergent validity and discriminant validity (see Table 8, Table 9, Table 10, Table 11 and Table 12).

The results of the CFA are presented in Table 8. All indicators loaded strongly on the corresponding latent constructs with the factor loadings ranging from 0.743 to 0.875 (all above 0.70, indicating high indicator reliability). The highest loading was obtained for the item S1 (satisfaction) of the construct satisfaction with λ = 0.875. The indicators of the memorability construct were also loaded strongly with factor loadings ranging from 0.771 to 0.851. Similar high loadings were obtained for the items of the construct learnability with factor loadings ranging from 0.795 to 0.838. The items of the efficiency construct were also loaded substantially with factor loadings ranging from 0.754 to 0.842.

Error handling showed the lowest loading of all study variables, with values between 0.743 and 0.770. However, all values are greater than the minimum required criterion and thus the indicators for this construct are reliable in capturing the perceptions of its respective dimensions error prevention, error recovery and system guidance. All freely estimated factor loadings were significant on the 0.001 level, which provides sufficient evidence that the indicators of the latent variables under study are adequate to measure what they are supposed to measure. Thus, all indicators of all dimensions can be retained for further analysis.

The results from the reliability and validity assessment for the measurement of the described constructs are displayed in Table 9. Concerning internal consistency reliability, the application of Cronbach’s alpha and Composite Reliability (CR) provided values between 0.797 and 0.862. All values exceed the threshold of 0.70 recommended for reliable results for all of the measured constructs. The highest value for the internal consistency was achieved for the construct learnability with a value of 0.862, which is only slightly higher than the values for memorability (0.856) and error handling (0.797).

The CR values of the measures ranged from 0.798 to 0.860 (all above 0.70). As before, the CR for learnability was the highest value (0.860); thus, it can be concluded that the measures for this factor are reliable as they form a good total indicator for the underlying construct. All other constructs have acceptable reliability values, thereby ensuring the use of reliable measures. This also supports the convergent validity of the measures by assessing the Average Variance Extracted (AVE) for each of the factors. The results for the Average Variance Extracted are provided in Table 12, and all of the factors have values greater than 0.50, with a high of 0.673 for learnability, followed by 0.665 for memorability. The lowest value for the Average Variance Extracted is 0.568 for error handling, but this is still greater than the recommended cutoff and thus indicates that all of the measures have good reliability and convergent validity.

### 4.6. Discriminant Validity and Second-Order Justification

The discriminant validity was assessed using the Fornell–Larcker criterion, which requires the square root of the Average Variance Extracted (AVE) for each construct to be greater than its correlations with the other constructs in the set. The diagonal elements in Table 10 represent the square roots of the AVE of each construct. The off-diagonal elements in the table represent the inter-construct correlations. The results of the correlations between the usability dimensions were extremely high. Correlations between learnability and memorability, between memorability and efficiency as well as between efficiency and learnability were all higher than the square roots of the AVE for the respective constructs. For instance, while the square root of the AVE for efficiency was 0.792, its correlation with memorability was 0.846. Correspondingly, the square root of the AVE for learnability was 0.820, while its correlation with memorability was 0.864.

While from a measurement perspective our findings do not satisfy the Fornell–Larcker criterion, they have, however, a meaningful explanation. Instead of indicating poor measurement quality, efficiency, learnability, memorability and error handling are four interrelated dimensions that describe a single usability experience in detail. These results are in line with the usability theories that have been developed by Nielsen and other experts on the topic, such as usability being composed of a set of complementary dimensions that together determine the user’s overall experience. However, the correlations between satisfaction and the usability dimensions were relatively low (0.781–0.797) and thus clearly below the Fornell–Larcker criteria. They only partly deviate from each other, however, and thus indicate that while clearly distinct from usability, the perceptions about it strongly affect users’ overall satisfaction. Thus, the results support our a priori definition of usability as a higher-order construct.

An additional assessment of the discriminant validity for the factors was conducted by calculating the Heterotrait–Monotrait ratio (HTMT) for all pairs of factors. Based on previous research, an HTMT ratio of less than 0.85 is strong, and less than 0.90 is acceptable for discriminant validity. The HTMT ratios for the usability measures were all above 0.929 and ranged from 1.007 to 1.014 for the pairs of first-order measures. The ratio for the pair of learnability and memorability was 1.007, the ratio for the pair of error handling and memorability was 1.014, and the ratios for efficiency and the other three usability measures were all close to or exceeded 1.0. Thus, there is substantial conceptual overlap between the first-order measures for the four usability dimensions.

Note that, unlike between construct relationships, high within-construct relationships do not suggest measurement failure. Rather, they support the second-order factor model that was at the heart of this research. High HTMT values indicate that the four user benefit constructs (efficiency, learnability, memorability, error handling) are not perceived to be completely distinct and that therefore they are best considered as four aspects of one overall usability experience. This view is strongly supported by Fornell–Larcker’s results, which found high inter-construct correlations as well as good reliability and convergent validity (see Table 10 and Table 11). The four usability dimensions, learnability, usability, memorability and efficiency, were furthermore modeled as first-order indicators of a higher-order construct of usability. The theoretical model of usability proposed by Nielsen and subsequent usability research was used as a basis. A second-order specification is adequate, since the structural model of the second-order specification of usability fits the observed data very well (Table 12). The CFI, TLI and GFI indicate perfect fit (values > 0.940), while the RMSEA indicates a very small error of fitting (value < 0.060). Thus, a higher-order factor of usability explains the four usability dimensions very well and the results are satisfactory and on a sufficient level of explanation. The evidence from Table 9, Table 10 and Table 11 supports strong indicator reliability, sufficient internal consistency, acceptable convergent validity, and considerable correspondence between the first-order measures of usability in the current study. The support for the conceptualization of usability as a second-order factor is sufficient to proceed with the analysis using structural equation modeling.

The results of the structural model are presented in Table 12. Consistent with H1, older age individuals’ mobile usage duration had a positive and significant effect on the usability of mHealth services for older age individuals (β = 0.616, SE = 0.087, z = 10.374, *p* < 0.001). The result implies that the more an individual uses his/her mobile, the more he/she would find mHealth services usable. The coefficient for usability and user satisfaction, β = 0.953, SE = 0.078, z = 14.585, *p* < 0.001, supports H2. The relationship between usability and user satisfaction was the strongest within the structural model, reinforcing the primary influence of usability upon the experience that older people have when using mHealth applications. User satisfaction was also positively related to use frequency (β = 0.193, SE = 0.024, z = 3.086, *p* = 0.002) for H3. The effect size of this relation was smaller than the one for the relation between usability and user satisfaction. However, older adults used mHealth applications more frequently when they reported higher levels of user satisfaction.

## 5. Discussion

This study examines the relationships between mobile usage duration, usability perceptions, user satisfaction and use of mHealth applications among older adults. Using a second-order structural equation model, the study empirically supports usability as the central mechanism of influence between older adults’ smartphone experience and their continued use of mHealth applications. Our study contributes to the growing body of the literature about mHealth adoption. The majority of studies that have explored the adoption of mHealth applications have concentrated on technology acceptance (TA) constructs such as perceived usefulness, perceived ease of use, attitude and users’ intentions. The recent study conducted by Farooq and Bashir (2025) [40] revealed that in order to enhance adoption of mHealth applications, perceived usefulness, perceived value, perceived ease of use, attitude towards adoption and users’ Fear of Missing Out (FOMO) should be increased. There are numerous psychological and motivational factors that affect the adoption of mHealth applications. In this study, we explored different aspects of usability and their associations with user satisfaction and mHealth application use among older adults. Thus, the study contributes to the existing body of knowledge on mHealth by establishing the significance of usability quality and user experience in sustaining users’ engagement post-adoption, particularly among older adults.

The findings of this study indicate that longer mobile usage by older adults would result in higher usability of mHealth applications. The findings also indicated that the efficiency, learnability, memorability and error handling of mHealth applications by older adults increased with the mobile usage duration. This is due to the fact that as older adults use mobile devices for longer periods of time, they become more proficient in using mobile devices to complete health-related tasks and to use mHealth applications efficiently. This finding supports the technology acceptance model findings that state prior experience with technology decreases cognitive barriers to using a new technology and increases a user’s confidence to use new digital health services. Recently, Chisty et al. (2025) reported the findings from their pilot study with older adults [41]. In this study, older adults reported that they often depend on their younger family members when using technology for completing tasks. Furthermore, they evaluated the usability of an LLM-enabled chatbot—designed using user-centered design (UCD) principles—for elderly users. The results showed significant improvement in usability outcomes for older adults following the intervention. Thus, it can be seen that both prior experience with technology use and age-friendly design are crucial for ensuring the effective adoption and subsequent use of mHealth by older adults.

The present study contributes to the research on usability by providing an empirical validation of the usability construct as a second-order construct. Similar to Wang et al.’s (2025) approach [42] to validate user experience as a higher-order multidimensional construct, the current study conceptualized the usability construct as consisting of four key dimensions, namely efficiency, learnability, memorability, and error handling. The measurement model yielded high reliability and validity for all of the mentioned dimensions. The second-order structural model furthermore revealed that the four dimensions collectively account for the usability construct as a whole.

Previous research into mHealth adoption by older adults has mainly dealt with issues related to technology readiness and the mHealth adoption intentions of older adults (Chiu et al., 2025) [43]. In this study, we have validated usability as a higher-order construct. Although efficiency, learnability, memorability, and error handling all had high reliability and were of high validity, the Fornell–Larcker and the HTMT results showed that they were very interrelated, and together they described the usability experience of older adults in more detail. In order to get a better insight into this experience, usability was modeled as a higher-order latent variable.

The results of this study support the well-known usability model of Nielsen, which presents usability as a multidimensional phenomenon. The strongest relationship in the structural model was between usability and user satisfaction (β = 0.953, *p* < 0.001). The significant impact that usability has on the mHealth user experience of older adults indicates that applications need to be easy to use, efficient while being used, and memorable over time. Our results are in line with prior human–computer interaction studies. They conceptualize usability as a multi-dimensional phenomenon and consider it as one of the most important factors for user satisfaction and overall user experience. According to Kivijärvi and Pärnänen (2021) [44], a satisfying user experience is created by a satisfying system usage, and several usability aspects create the impression of the system usage. Our results show strong intercorrelations between the usability dimensions. Hence, our results can be modeled by a higher-order latent variable for usability. This variable is influential for user satisfaction and for the continued usage of mHealth applications by older people.

Furthermore, applications also need to support the user during error recovery [45]. As older adults vary widely in their cognitive, perceptual, and technological abilities and needs, these factors significantly influence their perceptions of health technologies. Therefore, improving usability is essential for enhancing user satisfaction and increasing engagement with mHealth applications. In addition to the usability–satisfaction relationship, the results also support the positive effect of user satisfaction on use frequency (β = 0.193, *p* = 0.002). These findings support more recent evidence that user-centered design and user satisfaction are key determinants of continued engagement by older people with digital health technologies. Involving older people with digital health technologies in the design process can enhance the usability of these technologies and patient satisfaction with use of these technologies, thereby facilitating more effective and sustained use of health technologies by patients and their families (Murdock et al., 2026) [36]. Although this effect was somewhat smaller than the one observed for the relationship between usability–satisfaction and use frequency, the findings suggest that satisfied users are more likely to use mHealth applications frequently. Prior correlation and regression analyses indicated a very weak relationship between user satisfaction and usage frequency. However, SEM does account for measurement error and for relationships between latent variables. Therefore, SEM can capture relationships that would remain undetected in such prior analyses. This study complements the theoretical understanding of technology acceptance by the elderly from a perspective of an antecedent to their satisfaction and continuous use of mHealth services, namely of usability. Usability is a core characteristic of any technology, and in traditional TAM-based studies, it is mostly indirectly taken into account through such core determinants of technology acceptance as perceived usefulness and perceived ease of use. In the context of older adults’ mHealth services, however, a more detailed and integrated measure of usability, namely of a whole set of criteria such as efficiency, learnability, memorability, errors, and others, provides a more adequate account for their experiences with and perceptions of digital health services. Consequently, this study supports the merging of the theories of usability and technology acceptance. The above findings have practical implications. Firstly, when developing mHealth applications for older people, it is necessary to follow usability-oriented design principles. Interfaces should be simple, navigation should be easy to use, instructions clear, errors should be preventable and recovery support adequate. Healthcare providers and health policymakers that promote the use of digital health technologies among older people should realize that making health technologies more accessible is not sufficient to ensure long-term engagement. Usability of mHealth applications appears to play a significant role in their adoption, user experience and continued use of applications. By incorporating user-centered design into the development of mHealth applications, their usability can be improved, users’ experience and overall satisfaction can be increased, and people can become engaged in using digital health services in the long term.

## 6. Limitations

There are some limitations to the current study. The most important one is that this study adopted a cross-sectional approach; thus, it is difficult to establish causality between variables. Even though the structural model developed in this study is theoretically sound and statistically adequate, future longitudinal studies are required to confirm the order of mobile usage experience, usability, satisfaction and usage persistence.

The measurements were collected by means of self-reported survey questions. Although they are frequently used in usability engineering and in technology acceptance research, self-report instruments like the survey are afflicted by several types of bias, for example, by recall bias and by social desirability bias. Additionally, the interpretation of the survey questions on the part of the users can be different from the interpretation of the researchers. To obtain a more complete picture of the behavior of users, in the future, the survey has to be complemented by means of more objective behavioral measurements, for example, by application usage logs, by means of the duration of sessions, by means of navigation paths, by means of task completion rates and by real-time measurements of human–computer interaction.

A third limitation of the current study is that it was conducted amongst older adults in a specific national context. The study of healthcare technology usability therefore needs to take into account the specific healthcare systems, levels of digital literacy, the socio-economic environment and technology attitudes within a given country. Generalization to other populations or healthcare settings is therefore advised with caution. Replication of the model in other countries, and amongst younger as well as older adults, is recommended in order to assess the external validity of the current findings.

Additional aspects that influence users’ experiences and their intention to continue to use mHealth services in the future need to be integrated into the model. This could especially apply to aspects that have already been incorporated into other models of mHealth adoption. These would include aspects such as users’ trust, their concerns with regard to privacy, their views of security, their general health, their self-efficacy, their social networks and influences, the facilitating conditions through which they access mHealth services, and their digital health literacy. Future research could explore the relationships between these indicators and the indicators that are used in the model proposed here to measure the usability of mHealth services and the satisfaction that users feel with them.

The discriminant validity of the four antecedents to usability was substantial, yet to say, considerable. This is to say that there is sufficient empirical support for the higher-order factor representation of usability as presented in this paper. However, future research will be needed to more fully understand the potential antecedents of usability, and to explore whether or not the four factors presented in this study will subsume into yet other meaningful factors, such as the field of user characteristics and application domains.

## 7. Conclusions

The study explored the relationships between mobile usage duration, application usability, user satisfaction, and usage frequency of mHealth applications by older adults. The study integrated the well-established usability theory with established technology acceptance concepts. A second-order factor representing overall usability was validated including the four well-established usability aspects of efficiency, learnability, memorability, and error handling.

The results showed that increasing the amount of experience of using a smartphone was very important in enhancing older adults’ perception of the mHealth application’s usability. This variable was the most important variable affecting users’ satisfaction with the application. Users’ satisfaction with the application also affected the frequency of using the mHealth application. It was clear that positive experience with using a mHealth application would lead to continued use of the digital health application.

This study has several main contributions. Firstly, the study empirically tested the usability of mHealth systems as a higher-order construct. The individual usability dimensions (usefulness, flexibility, interface quality, and ease of use) showed good reliability and high convergent validity. The discriminant validity between the usability dimensions was also substantial, however, and supported a conceptualization of usability as a system of several interconnected dimensions that together form the usability experience of a user. The second-order structural equation model that described the usability experience of the participants had a very good fit and explained the mHealth adoption behavior of the older participants in a very effective way. From a practical perspective, the results of our study show that increasing the number of mobile devices in itself is not enough to ensure the successful implementation of mHealth applications. Application developers, healthcare providers, and policymakers need to focus on user-centered design in order to improve the usability of mHealth applications in general and especially for older people with a range of age-related cognitive, sensory or technological difficulties. Application design should include user-friendly navigation, clear instructions for use, effective error recovery mechanisms, and an intuitive interface in order to increase user satisfaction and guarantee continued use of the application.

Future studies could also be designed to incorporate a longitudinal perspective, an experimental usability evaluation, or multi-group SEM to study the development of usability perceptions in time, and the differences between various demographic groups. These additional studies would increase the evidence base that will be necessary to design and implement mHealth solutions of high usability that are accessible and sustainable for older people.

## Figures and Tables

**Table 1:** Participant selection and demographic characteristics of eligible participants.

Characteristic	Category	n	%
Survey responses received	Total responses	384	100.0
Excluded respondents	Age 50–59 years	113	29.4
Final eligible sample	Age ≥ 60 years	271	70.6
Age group	60–69 years	78	28.8
Age group	70–79 years	108	39.9
Age group	≥80 years	85	31.4
Gender	Male	125	46.1
Gender	Female	146	53.9
Educational level	Illiterate	107	39.5
Educational level	School education	96	35.4
Educational level	University degree	68	25.1

**Table 2:** Reliability analysis of study constructs.

Construct	Number of Items	Sample Size (N)	Cronbach’s Alpha
Efficiency	3	271	0.834
Learnability	3	271	0.862
Memorability	3	271	0.856
Error Handling	3	271	0.797
Satisfaction	3	271	0.835

**Table 3:** Descriptive statistics of study variables.

Variable	N	Mean	SD	Min	25%	Median	75%	Max	Skewness	Kurtosis
Efficiency	271	3.608	1.949	1.0	1.667	3.333	5.667	7.0	0.219	−1.371
Learnability	271	3.720	2.039	1.0	1.833	3.333	5.667	7.0	0.134	−1.359
Memorability	271	3.684	2.072	1.0	1.667	3.333	5.833	7.0	0.084	−1.438
Errors	271	3.755	1.956	1.0	2.000	3.667	5.667	7.0	0.113	−1.325
Satisfaction	271	3.857	1.875	1.0	2.333	3.333	6.000	7.0	0.161	−1.365

**Table 4:** Normality assessment of study variables.

Variable	N	Shapiro–Wilk W	*p*-Value	Skewness	Kurtosis
Efficiency	271	0.913	<0.001	0.219	−1.371
Learnability	271	0.911	<0.001	0.134	−1.359
Memorability	271	0.901	<0.001	0.084	−1.438
Errors	271	0.924	<0.001	0.113	−1.325
Satisfaction	271	0.915	<0.001	0.161	−1.365

**Table 5:** Pearson correlation matrix of study variables.

Variable	Mobile Usage	Efficiency	Learnability	Memorability	Error Handling	Satisfaction	Use Frequency
Mobile Usage	1.00	0.63 **	0.58 **	0.59 **	0.55 **	0.52 **	0.39 **
Efficiency	0.63 **	1.00	0.82 **	0.86 **	0.81 **	0.80 **	0.27 **
Learnability	0.58 **	0.82 **	1.00	0.84 **	0.83 **	0.79 **	0.24 **
Memorability	0.59 **	0.86 **	0.84 **	1.00	0.82 **	0.79 **	0.20 **
Error Handling	0.55 **	0.81 **	0.83 **	0.82 **	1.00	0.75 **	0.24 **
Satisfaction	0.52 **	0.80 **	0.79 **	0.79 **	0.75 **	1.00	0.11
Use Frequency	0.39 **	0.27 **	0.24 **	0.20 **	0.24 **	0.11	1.00

** indicates *p* < 0.01.

**Table 6:** Structural model path coefficients.

Hypothesis	Structural Path	β	SE	z-Value	*p*-Value	Result
H1	Mobile Usage Duration → Usability	0.616	0.087	10.374	<0.001	Supported
H2	Usability → User Satisfaction	0.953	0.078	14.585	<0.001	Supported
H3	User Satisfaction → Use Frequency	0.193	0.024	3.086	0.002	Supported

**Table 7:** Model fit indices for the second-order structural equation model.

Fit Index	Recommended Threshold	Obtained Value	Interpretation
χ^2^ (Chi-square)	Non-significant preferred	209.732	Reported
df (Degrees of Freedom)	—	115	Reported
χ^2^/df (Normed Chi-square)	<3.00	1.824	Excellent Fit
CFI (Comparative Fit Index)	>0.90 (Ideal ≥ 0.95)	0.972	Excellent Fit
TLI (Tucker–Lewis Index)	>0.90 (Ideal ≥ 0.95)	0.966	Excellent Fit
GFI (Goodness-of-Fit Index)	>0.90	0.940	Excellent Fit
AGFI (Adjusted Goodness-of-Fit Index)	>0.80	0.928	Excellent Fit
RMSEA (Root Mean Square Error of Approximation)	<0.08 (Ideal ≤ 0.06)	0.055	Excellent Fit
AIC (Akaike Information Criterion)	Lower is better	74.452	Parsimonious Model
BIC (Bayesian Information Criterion)	Lower is better	211.333	Parsimonious Model

**Table 8:** CFA factor loadings and indicator reliability.

Construct	Item	Standardized Loading (λ)	SE	z-Value	*p*-Value
Efficiency	E1	0.754	—	—	—
Efficiency	E2	0.776	0.077	13.459	<0.001
Efficiency	E3	0.842	0.081	14.819	<0.001
Learnability	L1	0.795	—	—	—
Learnability	L2	0.827	0.067	15.714	<0.001
Learnability	L3	0.838	0.066	16.023	<0.001
Memorability	M1	0.823	—	—	—
Memorability	M2	0.851	0.062	17.459	<0.001
Memorability	M3	0.771	0.063	15.031	<0.001
Error Handling	ER1	0.770	—	—	—
Error Handling	ER2	0.748	0.075	13.360	<0.001
Error Handling	ER3	0.743	0.072	13.252	<0.001
Satisfaction	S1	0.875	—	—	—
Satisfaction	S2	0.749	0.055	14.858	<0.001
Satisfaction	S3	0.745	0.054	14.732	<0.001

**Table 9:** Scale internal consistency and convergent validity.

Construct	Items	Cronbach’s Alpha (α)	Composite Reliability (CR)	Average Variance Extracted (AVE)	CR Assessment	AVE Assessment
Efficiency	3	0.834	0.834	0.627	Adequate	Adequate
Learnability	3	0.862	0.860	0.673	Adequate	Adequate
Memorability	3	0.856	0.856	0.665	Adequate	Adequate
Error Handling	3	0.797	0.798	0.568	Adequate	Adequate
Satisfaction	3	0.835	0.834	0.627	Adequate	Adequate

**Table 10:** Fornell–Larcker discriminant validity matrix.

Construct	Efficiency	Learnability	Memorability	Error Handling	Satisfaction
Efficiency	0.792	0.827	0.846	0.804	0.797
Learnability	0.827	0.820	0.864	0.837	0.795
Memorability	0.846	0.864	0.815	0.838	0.787
Error Handling	0.804	0.837	0.838	0.754	0.781
Satisfaction	0.797	0.795	0.787	0.781	0.792

**Table 11:** HTMT discriminant validity matrix.

Construct	Efficiency	Learnability	Memorability	Error Handling	Satisfaction
Efficiency	—	0.976	1.000	0.984	0.952
Learnability	0.976	—	1.007	1.009	0.934
Memorability	1.000	1.007	—	1.014	0.929
Error Handling	0.984	1.009	1.014	—	0.956
Satisfaction	0.952	0.934	0.929	0.956	—

**Table 12:** Structural model path coefficients for the second-order usability framework.

Structural Path	β	SE	z-Value	*p*-Value
Mobile Usage → Usability	0.616	0.087	10.374	<0.001
Usability → Satisfaction	0.953	0.078	14.585	<0.001
Satisfaction → Use Frequency	0.193	0.024	3.086	0.002

## Data Availability

The data presented in this study are available on request from the corresponding author. The data are not publicly available due to privacy and ethical restrictions.

## Appendix A. Survey Questionnaire Items

**Table A1:** Measurement instrument.

Construct	Item Code	Item Statement
Efficiency	E1	I can complete health-related tasks quickly using the application
Efficiency	E2	The application helps me achieve my health goals with minimal effort
Efficiency	E3	The steps required to perform tasks are efficient and organized
Learnability	L1	It is easy to learn how to use the application
Learnability	L2	I can use the main features without external help
Learnability	L3	Instructions and interface are clear and understandable
Memorability	M1	I can easily remember how to use the application
Memorability	M2	The design helps me recall how to perform tasks
Memorability	M3	I do not need to relearn the application each time
Errors	ER1	I rarely make errors while using the application
Errors	ER2	The application clearly explains how to fix errors
Errors	ER3	Error messages help me recover quickly
Satisfaction	S1	I feel comfortable using the application
Satisfaction	S2	I am satisfied with my overall experience
Satisfaction	S3	The application gives me confidence in managing my health

## Appendix B. Statistical Details

**Table A2:** Normality assessment.

Variable	Skewness	Kurtosis	Interpretation
Mobile Usage	0.108	−1.551	Approximately Normal
Efficiency	−0.222	−1.496	Approximately Normal
Learnability	−0.321	−1.365	Approximately Normal
Memorability	−0.412	−1.344	Approximately Normal
Error Handling	−0.358	−1.327	Approximately Normal
Satisfaction	−0.286	−1.438	Approximately Normal
Use Frequency	0.236	−1.957	Approximately Normal

**Table A3:** Regression diagnostics (multicollinearity).

Predictor	VIF	Interpretation
Efficiency	2.12	Acceptable
Learnability	2.34	Acceptable
Memorability	2.28	Acceptable
Error Handling	1.89	Acceptable

### Appendix B.1. Regression Analysis

This initial assessment of the study variables’ ability to predict one another was conducted via a series of regression models prior to their employment within a structural equation model. Table A4 contains summaries of the respective regression models, and full details of each regression’s coefficients are contained within Table A5.

The results for predicting the usability dimensions of usability indicate that mobile usage duration is a significant predictor of all three dimensions. In terms of the amount of explained variance by the regression models, efficiency was found to be explained by 35.2% of the variance (R^2^ = 0.352), learnability by 32.2% of the variance (R^2^ = 0.322), and memorability by 32.7% of the variance (R^2^ = 0.327). All of the models were statistically significant at *p* < 0.001. The results for predicting user satisfaction from the usability dimensions indicated that a substantial amount of variance was explained by the model, with 71.4% of the variance explained by satisfaction (R^2^ = 0.714, Adjusted R^2^ = 0.709). The F-statistic for the model was highly significant at *p* < 0.001, indicating that the model performed well in terms of overall prediction.

Table 7 shows the results of the detailed regression analysis. For the user satisfaction model, efficiency (β = 0.296, *p* < 0.001), learnability (β = 0.218, *p* = 0.002), and error handling (β = 0.215, *p* = 0.001) all positively predicted satisfaction. Memorability had a positive coefficient (β = 0.121) but did not reach significance (*p* = 0.084). Therefore, in addition to task efficiency, users consider an mHealth application to be easy to use when they can complete a task quickly and have a positive experience if they encounter errors and if they are also satisfied with their overall use of the application.

**Table A4:** Summary of regression models.

Model	R^2^	Adjusted R^2^	F-Statistic	*p*-Value	N
Model 1: Efficiency	0.352	0.350	146.336	<0.001	271
Model 2: Learnability	0.322	0.320	128.038	<0.001	271
Model 3: Memorability	0.327	0.325	130.817	<0.001	271
Model 4: Satisfaction	0.714	0.709	165.759	<0.001	271

The regression model of user satisfaction against use frequency has a positive but non-significant coefficient of 0.032 (*p* = 0.188). Hence, no direct significant relationship between user satisfaction and use frequency was found in the regression analysis. The relationship between user satisfaction and use frequency was further analyzed with the help of structural equation modeling, which is the main methodology of this research.

**Table A5:** Regression analysis results for user satisfaction and use frequency.

Target Construct	Predictive Path	β	SE	t-Value	*p*-Value	Result
User Satisfaction	Efficiency	0.296	0.065	4.565	<0.001	Significant
	Learnability	0.218	0.068	3.198	0.002	Significant
	Memorability	0.121	0.070	1.732	0.084	Not Significant
	Error Handling	0.215	0.065	3.323	0.001	Significant
Use Frequency	User Satisfaction	0.032	0.024	1.320	0.188	Not Significant

### Appendix B.2. Mediation Analysis

An additional investigation of the indirect effects on user satisfaction was conducted using a bootstrapped mediation analysis, with the results presented in Table A6.

**Table A6:** Mediation analysis results (bootstrapping).

Mediator	a Path	b Path	Indirect Effect	95% CI Lower	95% CI Upper
Efficiency	1.028	0.753	0.774	0.626	0.939
Learnability	1.029	0.702	0.722	0.592	0.854
Memorability	1.053	0.682	0.718	0.583	0.867
Error Handling	0.912	0.694	0.633	0.511	0.760

All four usability dimensions were found to significantly mediate the relationship between mobile usage duration and user satisfaction. The largest indirect effect was found for efficiency (Indirect Effect = 0.774; 95% CI [0.626; 0.939]), followed by learnability (Indirect Effect = 0.722; 95% CI [0.592; 0.854]), memorability (Indirect Effect = 0.718; 95% CI [0.583; 0.867]) and error handling (Indirect Effect = 0.633; 95% CI [0.511; 0.760]).

For all of the mediators, the 95% bootstrapped confidence intervals did not contain zero, indicating that there were statistically significant mediation effects. The positive effect of the experience that users gained from using their smartphones on their satisfaction with mHealth applications was through their perceptions of the usability of mHealth applications. Users of mHealth applications who have had experience with using smartphones have very positive perceptions of the efficiency of mHealth applications, the ease with which they can learn to use mHealth applications, how easily they can remember how to use mHealth applications, and the ease with which mHealth applications recover from errors. Overall, their satisfaction with mHealth applications is very high. Of the usability aspects considered, efficiency had the largest indirect effect, which, therefore, is also the largest effect that smartphone experience has on user perceptions of mHealth applications. Overall, the mediation results therefore provide further support for the usability-centered framework proposed here and illustrate the central position that users’ perceptions of usability take in determining their satisfaction with mHealth applications for older adults.

## Appendix C. Instrument Development and Validation

This survey tool was constructed based on a host of empirical studies on usability and technology acceptance by end users, especially with respect to older adults and mHealth applications. The survey consisted of measures for efficiency, learnability, memorability, error handling, and user satisfaction with the system.

To establish content validity for the survey instrument, a panel of experts consisted of health informatics specialists, digital health specialists and usability specialists. They assessed all items in terms of clarity, relevance and appropriateness for the intended constructs and for older users. Minor changes to the items were made in order to make wording more adequate, and to check for cultural appropriateness.

A pilot study was performed to test the comprehensibility of the questions of the survey as well as the length of the survey and the format of answers. Furthermore, the functionality of the electronic survey was checked. The pilot study consisted of a small group of older adults. They were able to complete the questionnaire without difficulties being reported. Minor changes to wording were made to improve readability. The data of the pilot study were not included in the analysis of the study results. The results of the pilot study proved that the survey was suitable for the purposes of the main study.
